# Emerging Roles of Metal–Organic Frameworks as Flame Retardants: Recent Advances and Future Perspectives in Thermoplastic Polymers

**DOI:** 10.3390/ma19010150

**Published:** 2025-12-31

**Authors:** Jiayi Ding, Zihan Zhang, Zhi Wang, Yichao Lin, Ye-Tang Pan, Kun Yao

**Affiliations:** 1 National Engineering Research Center of Flame Retardant Materials, School of Materials Science and Engineering, Beijing Institute of Technology, Beijing 100081, China; 2 State Key Laboratory of Coal Mine Disaster Prevention and Control, School of Safety and Engineering, China University of Mining and Technology, Xuzhou 221116, China; 3 Guangdong Advanced Carbon Materials Co., Ltd., Zhuhai 519000, China

**Keywords:** metal–organic framework (MOF), thermoplastic, flame-retardant, flame-retardant mechanism

## Abstract

**Highlights:**

**What are the main findings?**

- Flame-retardant metal-organic frameworks (MOFs) have attracted growing interest in polymer applications, with substantial research progress achieved.
- Studies on thermoplastic polymers, despite their widespread use and large consumption, remain relatively limited and comprehensive reviews are still scarce.
- Six representative thermoplastic matrices have been identified for evaluations: polypropylene (PP), thermoplastic polyurethane (TPU), polylactic acid (PLA), polycarbonate (PC), polyethylene terephthalate (PET), and polystyrene (PS).
- The current research status of flame-retardant MOFs in these thermoplastic polymers is systematically summarized and analyzed.
- Existing challenges and future development prospects are discussed to provide guidance for the design and development of MOF-based flame retardants.

**What are the implications of the main findings?**

- Flame-retardant MOF plays an alternative to traditional retardants.
- Thermoplastic application gap identification fills knowledge void, guiding academia and industrial translation.
- Summary of six thermoplastics guides research, accelerating commercialization.

**Abstract:**

Metal–organic frameworks (MOFs), assembled from inorganic metal centers (metal ions or clusters) and organic ligands, possess distinctive features such as structural designability, high surface area, and tunable functionalities. In the past decade, MOFs have displayed substantial merits when utilized as innovative flame retardants in the realm of polymeric materials. A current focus is on the flame-retardant effects of MOFs in thermosetting plastics, yielding substantial achievements; however, systematic investigations into thermoplastic polymers, which are more widely used, remain limited. The flame-retardant mode of action for miscellaneous types of MOFs and their applications in polymeric matrices, with particular emphasis on recent advances in thermoplastic systems, are summarized. Furthermore, existing challenges and future perspectives are identified.

## 1. Introduction

Polymeric materials, particularly thermoplastics such as polypropylene (PP), thermoplastic polyurethane (TPU), polylactic acid (PLA) and poly-carbonate (PC), are indispensable in modern industries including the electronics, automotive, construction, and textile industries, owing to their light weight, processability, and exceptional mechanical properties [1,2,3,4]. However, their intrinsic flammability remains a critical limitation, as it can induce severe fire hazards under accidental ignition conditions—including thermal overload, electrical short circuits, or external flame exposure—in various industrial and civilian applications, potentially resulting in substantial loss of life and property [5,6]. Consequently, controlling the flammability of thermoplastic polymers has been a long-standing challenge and a major focus in materials science. Halogenated compounds and traditional flame retardants (FRs) such as nitrogen- and phosphorus-based additives were once widely employed due to their relatively high efficiency and low cost. However, increasing societal concern regarding environmental impact, together with the release of toxic and corrosive gases during thermal decomposition, has led to stringent regulation and the gradual phase-out of several conventional FRs [7,8,9,10].

MOFs, composed of metal ions or clusters coordinated with organic ligands, represent a class of crystalline porous materials. In recent years, their use as flame-retardant additions in polymer systems has gained significant momentum [11,12]. The unique features of MOFs that influence combustion, including structural tunability, high surface area, controllable porosity, and the ability to generate metal oxides or catalytically active carbonaceous residues upon thermal decomposition, confer strong potential for promoting char formation and enhancing thermal stability [13,14,15,16,17,18,19].

**Figure 1:**
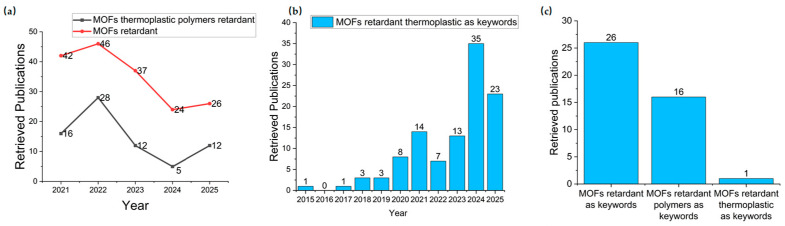
(**a**) The number of retrieved publications in the past five years searched by different keywords in the Web of Science (WOS) database as of 8 November 2025. (**b**) The proportion of the applications of flame-retardant MOFs in thermoplastic polymers among their total applications in all polymers in the WOS database as of 8 November 2025. (**c**) The proportion of the number of review articles retrieved from WOS using “MOFs retardant”, “MOFs retardant polymer” and “MOFs retardant thermoplastic” as keywords, as of 8 November 2025.

Most evaluations of flame-retardant MOFs have been conducted in thermosetting systems such as epoxy resins (EP), polyurethane acrylate (PUA), and unsaturated polyesters (UPR), where MOFs exhibit quite attractive performance in terms of flame retardancy and smoke suppression [20,21,22,23,24,25]. However, as shown in Figure 1, an enhancing search using different keywords in the WOS database as of 8 November 2025 reveals two key observations. On one hand, the number of research and review papers on flame-retardant MOFs has remained relatively high over the past five years, and the number of research papers focusing on MOFs for flame retardancy in thermoplastic matrices has increased rapidly over the past decade. This indicates that systematic research on thermoplastic polymers, which account for a large proportion of industrial and consumer polymers has become increasingly important in the research community and is attracting more and more engagement in this field. On the other hand, the use of MOFs for flame retardancy in thermoplastic matrices is still relatively limited. Therefore, a comprehensive overview of the role of MOFs as flame retardants in thermoplastics is provided. A summary of the latest progress on the use of MOFs for flame retardancy in thermoplastic polymers, classified according to the matrix materials in which MOFs are applied for flame retardancy, is provided. In addition, the great potential of MOFs in current flame-retardant applications as well as the challenges hindering their practical application and for use in prospects for future development are projected. A clear and understandable summary of the development of flame-retardant MOFs for use in thermoplastic systems is projected. Insightful references for the rational design and development of high-performance, sustainable, and low-cost MOF-based flame retardants are provided.

## 2. Flame-Retardant Mode of Action for MOFs

Combustion is inseparable from three essential elements, known as the “fire triangle”: oxygen, fuel, and a heat source or ignition source [26,27,28]. Owing to their structural characteristics, high-molecular polymers exhibit a complex process involving both the condensed and gas phases. At sufficiently high temperature, the polymer relies on the external “fire triangle” [29]. Naturally, the flame-retardant mechanism of MOFs is also inseparable from these aspects.

At elevated temperatures, polymers begin to undergo thermal degradation, generating small fuel fragments that are released into the gas phase. There, they mix with oxygen and, if the temperature reaches or exceeds the ignition point, combustion occurs. The process predominantly takes place in the gas phase above the polymer surface, where volatile pyrolysis products interact with oxygen to sustain flame propagation. The rate of formation and chemical composition of these volatiles critically determine flame intensity and spread, highlighting the pivotal role of gas-phase reactions in the overall flammability of polymers [30].

**Figure 2:**
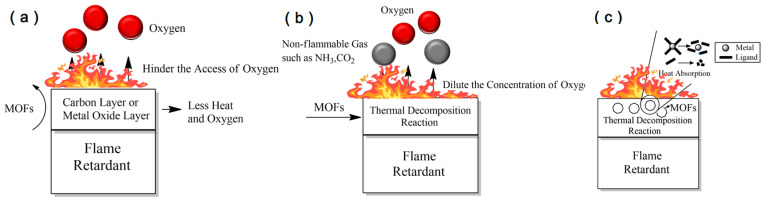
(**a**) MOF-catalyzed carbon layer (**b**); MOF gas-phase dilution (**c**); MOF endothermic heat absorption.

As shown in Figure 2, first, the physical barrier effect is the most direct flame-retardant pathway for MOFs. During combustion, on the polymers’ surface, the metal nodes in MOFs catalyze cross-linking, aromatization, and carbonization of polymer chains, promoting the formation of dense, continuous, and mechanically robust carbon layers that effectively insulate against heat and oxygen. At elevated temperatures, MOFs also undergo pyrolysis to generate metal oxide–carbon composite particles, further reinforcing the protective properties of the carbon layer. The dense layer effectively prevents the transfer of the heat from the combustion zone and reduces the rate of polymer pyrolysis and combustible fuel formation within MOFs. For example, Cu and Cu-oxides accelerate the generation of a carbon layer at the surface of the desorbing polymer. This carbon layer can hinder the transfer of heat from the combustion zone, thus slowing degradation of the polymer [31].

Second, the gas-phase dilution effect serves a remarkable role in the flame retardancy of MOFs. This effect mainly stems from the inert or diluting gases (such as CO_2_, NH_3_, N_2_, etc.) released by the pyrolysis of organic ligands in the MOF structure. These gases can form a local diluted atmosphere in the combustion zone, thereby reducing the effective concentrations of flammable gases and oxygen and inhibiting flame propagation. A Co-MOF has been utilized in polylactic acid (PLA). During thermal decomposition of Co-MOF and PLA, non-flammable gases are released, producing a dilution effect [18].

Finally, the flame-retardant effect induced by the intrinsic transformations of MOFs should not be overlooked. The endothermic decomposition of MOFs primarily involves the thermal degradation of organic ligands and the cleavage of coordination bonds between metal ions and organic ligands. In addition, some MOFs contain crystallized water, which can undergo endothermic desorption. MOFs are often capable of providing an initial heat-absorbing effect during the early stages of combustion, thereby delaying the temperature rise in the polymer matrix, lowering the surface temperature, postponing thermal degradation and the release of flammable gases, and ultimately slowing down flame propagation.

In practical applications, MOFs generally exert their flame-retardant effects through the synergistic action of these mechanisms. Future research should focus on controlling MOF dispersion within polymer matrices, enhancing interfacial compatibility, and balancing thermal stability with sustainability, in order to develop efficient and environmentally friendly flame-retardant systems.

## 3. MOFs in Thermoplastic Matrices for Fire Retardancy

Thermoplastic polymers constitute a major class of materials in modern industry and are widely applied owing to their favorable mechanical properties, recyclability, ease of processing, and excellent insulation performance [32,33,34,35]. However, most used thermoplastic polymers, including polypropylene (PP), polylactic acid (PLA), polycarbonate (PC), polyamide (PA), and thermoplastic polyurethanes (TPU), are inherently flammable. In addition, these materials release substantial amounts of heat and toxic gases during combustion, which severely limits their safe use in engineering applications such as electronics and electrical devices, automotive components, and construction materials [27,36]. For example, PLA exhibits a limiting oxygen index (LOI) of only 18–20%, indicating high flammability even under ambient oxygen conditions. This characteristic presents considerable fire hazards in practical applications such as additive manufacturing and 3D printing [37]. In recent years, MOFs, a class of porous materials with tunable structures, high specific surface area, and good thermal stability, have attracted increasing attention for flame-retardant modification of polymers. Although research on MOFs for improving the flame retardancy of thermoplastic polymers remains at an early stage, significant progress has been achieved in a variety of polymer matrices, highlighting their potential as a promising direction for the development of advanced flame-retardant materials.

Thermoplastic polymers constitute a major class of materials in modern industry and are widely applied owing to their favorable mechanical properties, recyclability, ease of processing, and excellent insulation performance [32,33,34,35]. However, most commonly used thermoplastic polymers, including PP, PLA, PC, PA and TPU, are inherently flammable. Moreover, these materials release substantial amounts of heat and toxic gases during combustion, which severely limits their safe use in engineering applications such as electronics and electrical devices, automotive components, and construction materials [27,36]. For instance, PLA exhibits a LOI of only 18–20%, indicating high flammability even under ambient oxygen conditions. This characteristic poses significant fire hazards in practical applications such as additive manufacturing and 3D printing [37]. In recent years, MOFs, a class of porous materials with tunable structures, high specific surface area, and good thermal stability, have attracted increasing attention for flame-retardant modification of polymers. Although research on MOFs for improving the flame retardancy of thermoplastic polymers remains at an early stage, significant progress has been achieved in a variety of polymer matrices, highlighting their potential as a promising direction for the development of advanced flame-retardant materials.

**Figure 3:**
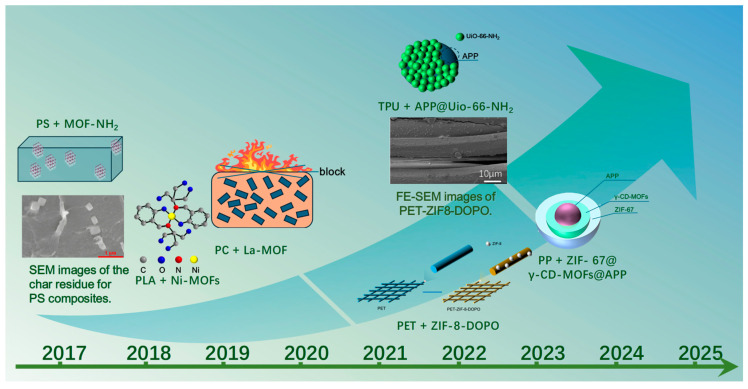
Representative examples of flame-retardant MOFs used in six major thermoplastic matrices and their first-reported timeline. Some subfigures in this illustration are directly reproduced from Refs. [38,39] and are labeled in accordance with the relevant copyright requirements.

It is generally acknowledged that the application of flame-retardant MOFs in polymers emerged around 2017. As shown in Figure 3, the first reported use of MOFs as flame-retardant additives in thermoplastic polymers was conducted in a PS matrix in 2017, followed by studies involving PLA and PC matrices in 2018 and 2019, respectively. With continued advances in this area, investigations expanded to polyethylene terephthalate PET and TPU matrices in 2022, and in 2023, further efforts focused on PP, one of the most widely used engineering plastics. In recent years, research on flame-retardant MOFs in thermoplastic polymers has grown rapidly. Meanwhile, it can be observed that around 2017, MOFs were primarily employed as single-component flame-retardant additives, whereas more recent studies have increasingly incorporated MOFs into synergistic flame-retardant systems in combination with other flame retardants. This transition reflects an evolving strategy in the development of MOF-based flame retardants. The following sections provide a systematic overview of these applications across six major thermoplastic polymer matrices.

### 3.1. MOFs in Polypropylene (PP) for Fire Retardancy

Polypropylene (PP) accounts for approximately 21% of global plastics production and is the most widely used thermoplastic polyolefin in daily applications [40,41]. However, PP exhibits a low LOI of approximately 17% and is prone to melting, deformation, and dripping during combustion, accompanied by the release of toxic gases. These limitations severely restrict its use in construction, automotive, home appliance, and packaging industries, while posing significant risks to human safety and property [42]. Although traditional inorganic flame retardants and IFR systems can enhance the flame retardancy of PP, and substantial progress has been achieved in their research and application, these approaches often require high loading levels and result in deterioration of mechanical properties [43]. As an emerging class of flame retardants, MOFs have attracted initial attention in PP systems; however, their application in this area remains at an early stage. Recent research subjects and their corresponding findings are presented in Table 1.

**Table 1:** Test data of flame-retardant performance of polypropylene (PP) with different MOFs.

**MOF Flame-Retardant System**	**Flame-Retardant Results**	**References**
IFR/ZIF-67	33.1%, 33.8%, 31.0% and 21.1% reduction in pHRR, THR, pSPR and TSP; 31.1% of LOI; UL-94 V-0	[44]
ZIF-67@MMT	For a PP composite with an IFR content of 25 wt% and ZIF-67@MMT content of 3 wt%, 93%, 52%, 94%, 86%, and 66% reduction in pHRR, THR, pSPR, TSP, and CO production; 32.1% of LOI; UL-94 V-0	[45]
ZIF-67@γ-CD-MOFs@APP	52.65%, 24.72%, 36.49%, and 52.29% reduction in PHRR, PSPR, CO production and CO_2_ production; 36.2% of LOI; UL-94 V-0; 12.47% of char residue	[46]
NH_2_-MIL-53 (Al)	88.55% and 79.75% reduction in pHRR and THR; 31.1% of LOI; UL-94 V-0	[47]

**Figure 4:**
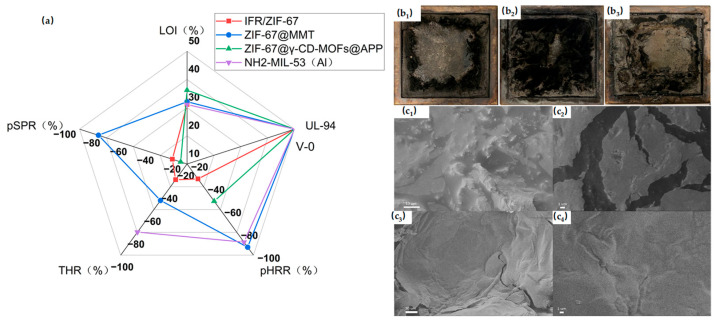
(**a**) Test data of pure IFR/ZIF-67, ZIF-67@MMT, ZIF-67, ZIF-67@γ-CD-MOFs@APP and NH_2_-MIL-53 (Al). (**b_1_**–**b_3_**) Digital photographs of combustion residues of (**b_1_**) pure PP, (**b_2_**) PP with 1% MOFs and (**b_3_**) PP with 1% MOFs after cone calorimeter tests; (**c_1_**–**c_4_**) SEM images of the char residue’s PP without ZIF-8 (**c_1_**,**c_2_**) and PP with 2% ZIF-8 (**c_3_**,**c_4_**) after cone calorimeter tests. Reproduced with permission from ref. [50].

Figure 4 shows the performance comparison of the research results. Zeolitic Imidazolate Framework (ZIF) materials belong to a subclass of MOFs. They form a tetrahedral framework structure through the connection between transition metal atoms and imidazole or imidazole derivatives, exhibiting high stability, high porosity, and organic functional properties [43,48]. Among them, transition metal-based MOFs (such as ZIF-8(Zn) and Co-based ZIF-67(Co)) demonstrate excellent dispersibility, smoke suppression, and catalytic carbonization capabilities in PP [43]. This is attributed to the transition metal (Co) present in ZIF-67, which offers abundant catalytic sites during combustion, promoting dehydration and charring, and consequently enhancing the graphitization degree of the resulting carbon layer. Currently, mainstream ZIFs do not act alone on PP to achieve flame retardancy; instead, they form flame-retardant systems by synergizing with flame retardants such as IFR and hexachlorocyclotriphosphazene (HCCP), or by doping with other metal atoms. For instance, ZIF-67 was prepared and incorporated as a synergistic additive into PP composites containing an IFR system, resulting in improved flame retardancy and enhanced smoke suppression performance. The IFR/ZIF-67 composite flame-retardant system showed outstanding flame-retardant modification in vertical burning tests [44].

To modify the microstructure of ZIFs and optimize their flame-retardant performance, ZIF-67 was grown in situ on the surface of montmorillonite (MMT) layers, resulting in a heterogeneous composite structure denoted as ZIF-67@MMT. Microstructural observations revealed that ZIF-67@MMT possesses superior high specific surface area and high dispersibility in polymers, which accounts for its outstanding flame-retardant performance. PP-based composites containing 25 wt% IFR and 3 wt% ZIF-67@MMT exhibited a LOI of 32.1% and achieved a UL-94 V-0 rating [45]. Considering the synergistic effect of cyclodextrins, phosphorus-containing flame retardants, and MOFs, Wu et al. compounded ZIF-67, γ-cyclodextrin-based MOFs (γ-CD-MOFs), and ammonium polyphosphate (APP) into ZIF-67@γ-CD-MOFs@APP, which was then applied to PP. This composite also synergistically enhanced the flame retardancy and smoke suppression properties of PP [49]. Leveraging the complementary interactions among cyclodextrins, phosphorus-containing flame retardants, and MOFs, a hybrid additive was designed. ZIF-67,γ-CD-MOFs and APP were combined to form a hybrid additive (ZIF-67@γ-CD-MOFs@APP), which was subsequently incorporated into PP. This system produced a synergistic enhancement in flame retardancy and smoke suppression performance.

Materials of Institute Lavoisier (MIL) series MOFs also find applications in PP flame-retardant materials. NH_2_-MIL-53(Al) was synthesized and integrated into PP. The resulting composite exhibited an LOI of 31.1% and achieved a UL-94 V-0 rating, indicating a remarkable enhancement in flame retardancy and thermal stability, while mechanical tests showed no significant loss in properties [47]. Furthermore, the combined use of MOFs and phosphorus-containing flame retardants (e.g., APP) has been found to produce a significant synergistic effect. This synergy is generally manifested in two aspects: in the condensed phase, the phosphorus fraction forms a porous char residue, and MOFs promote the densification of the polymer char layer structure. Additionally, APP, a commonly used flame retardant, can release NH_3_ during thermal decomposition, and this synergistic effect significantly improves its flame-retardant performance. In current research on polymer flame retardants, such MOF/phosphorus composite systems can usually balance both flame retardancy efficiency and the retention of polymer material mechanical properties. This demonstrates the great potential of MOFs for enhancing flame retardancy in thermoplastic polymers with high mechanical property requirements.

### 3.2. MOFs in Thermoplastic Polyurethane (TPU) for Fire Retardancy

TPU is a unique class of polymers that can be described as an (AB)_n_-type linear block copolymer. In this structure, segment A typically consists of high-molecular-weight polyester or polyether soft segments, while segment B is derived from diols containing a certain number of linear aliphatic carbon atoms, forming the hard segments. Owing to its excellent compatibility, outstanding mechanical properties, tunable elasticity, and recyclability, TPU has secured a significant share in the thermoplastic polymer market. As a result, TPU has been widely applied in diverse fields, including automotive components, footwear materials, flexible tubing and hoses, wire and cable jacketing, medical devices, wearable electronics, and protective coatings [25,51,52,53]. Recent research subjects and their corresponding findings are presented in Table 2.

Zr-based MOFs (e.g., UiO-66) are widely used for flame-retardant modification of polymers due to their excellent thermal stability and high specific surface area, which can reduce heat release and significantly suppress smoke generation. Upon combustion, transition-metal-based MOFs like Zr-MOFs convert into metal oxides, increasing the char layer density and enhancing its physical barrier effect. UiO-66(Zr) and NH_2_-UiO-66(Zr) were prepared and further functionalized with SiO_2_ to enhance the flame retardancy and smoke suppression of TPU. Even at a low loading of 2%, both MOFs significantly increased the LOI and improved the UL-94 rating. Notably, SiO_2_-functionalized NH_2_-UiO-66(Zr) exhibited superior performance, as the formed zirconium oxide and carbonized residues restricted contact between combustible solids and flammable gases, thereby delaying ignition [54].

Additionally, NH_2_-UiO-66(Zr) can release nonflammable nitrogen gas, which dilutes the concentration of flammable volatiles. These combined effects demonstrate that functionalized Zr-MOF composites can serve as highly effective flame retardants in TPU [54]. Shi et al. further investigated the potential synergistic behavior between MOFs and inorganic phosphorus-containing flame retardants. They employed the common Zr-based MOF UiO-66-NH_2_(Zr) to modify APP, yielding a synergistic flame-retardant system denoted as APP@MOFs. The resulting TPU/APP@MOFs composites displayed excellently in a series of combustion tests [55]. Their findings clearly indicate that the inherent structural characteristics of MOFs impart catalytic and synergistic functionalities that significantly enhance the catalytic charring and gas-phase dilution effects of APP within PLA matrices. Simultaneously, ZIF–type MOFs have also been widely applied in TPU matrices [61].

Structural features strongly influence performance. By adjusting the 2-methylimidazole to Co(II) nitrate hexahydrate ratio and introducing an appropriate amount of the surfactant CTAB during synthesis, three distinct ZIF-67 morphologies were obtained: dodecahedral, cubic (ZIF-67 NC), and cruciform (ZIF-67 H). Characterization revealed that ZIF-67 H exhibited superior performance compared with the other two morphologies [56]. In flame-retardant design, multilevel and multicomponent architectures generally outperform traditional single-structure systems. A nano-hybrid (ZIF-67H@PBA) was thus constructed using ZIF-67 H as the host and PBA as the guest MOF, forming a unique core–shell, three-dimensional cross-linked heterostructure. TPU/ZIF-67H@PBA composites at ultralow loadings exhibited excellent flame retardancy, along with effective smoke suppression and reduced toxicity [62]. Core–shell architectures represent a promising strategy for designing hierarchical MOF-based flame retardants.

In addition, MXenes are a class of two-dimensional inorganic materials considered highly promising functional nanofillers due to their excellent flexibility, electrical conductivity, intrinsic flame retardancy, and layered structures that facilitate uniform dispersion within polymer matrices. In TPU flame-retardant systems, synergistic combinations of MOFs and MXenes have been extensively investigated [63,64,65]. In practical flame-retardant applications, the intrinsic structural tunability of MOFs enables diverse interactions with other materials, thereby modifying their properties and offering broad opportunities for exploration. Ni-MOFs were functionalized on multi-terminal MXene (Ti_3_C_2_T_x_) and applied for flame retardancy in TPU. The Ni-MOF@Ti_3_C_2_T_x_, prepared via a solvothermal method, exhibited notable flame-retardant performance at a loading of only 1 wt% in TPU composites, particularly showing a remarkably high char yield [57]. The incorporation of MOFs enhanced the dispersibility, thermal stability, and flame-retardant performance of MXene. MOFs can thus serve not only as primary flame retardants but also as synergistic additives, broadening design strategies and application possibilities for MOF-based flame-retardant systems.

**Figure 5:**
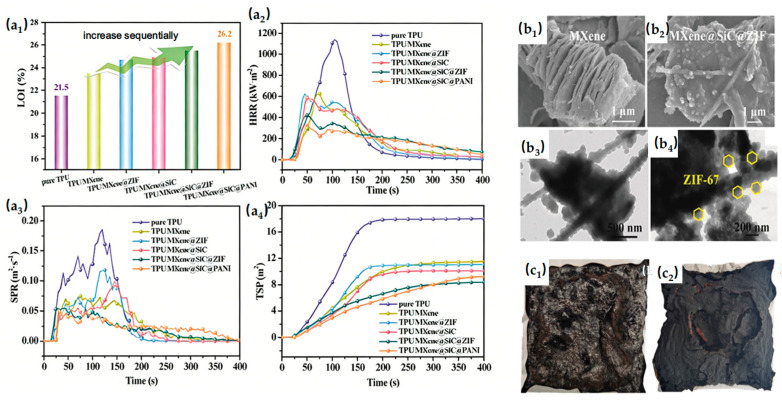
(**a_1_**–**a_4_**) Test data of pure TPU, TPU/MXene, TPU/MXene@ZIF, TPU/MXene@SiC, TPU/MXene@SiC@ZIF and TPU/MXene@SiC@PANI: (**a_1_**) LOI values, (**a_2_**) HRR; (**a_3_**) SPR; (**a_4_**) TSP. (**b_1_**–**b_4_**) (**b_1_**,**b_2_**) SEM image of MXene and MXene@SiC@PANI; (**b_3_**,**b_4_**) TEM image of MXene@SiC@ZIF, reproduced with permission from ref. [59]. (**c_1_**,**c_2_**) The digital combustion pictures: (**c_1_**) TPU/10SiAPP-NH_2_@1MOFs, (**c_2_**) TPU/1SiAPP-NH_2_@1MOFs. Reproduced with permission from ref. [60].

Modifying MXene with MOFs is an effective strategy to overcome its limitations. MXene alone tends to aggregate in polymer matrices, compromising mechanical properties. A lanthanide-based MOF (La-MOF) was grown in situ on MXene surfaces to form La-MOF@MXene. In this system, La-MOF suppressed the aggregation of MXene, and the synergistic interaction improved the uniformity of flame-retardant dispersion in TPU, maintaining mechanical integrity. At a loading of 3 wt%, TPU/La-MOF@MXene composites exhibited improvements across multiple combustion indices [58]. To address MXene’s intrinsic challenges, including poor delamination and agglomeration in matrices, ZIF-67 and SiC were loaded in situ onto MXene, followed by in situ formation of polyaniline (PANI) on the ZIF-67 surface. The resulting TPU/MXene@SiC@PANI system exhibited enhanced performance in combustion test [59]. Figure 5 demonstrates the achievements in terms of test data of TPU with different MOFs. These studies offer strategies for developing hybrid flame retardants combining MOFs and two-dimensional MXene nanosheets, expanding applications and enhancing the flame-retardant performance of both components.

Emerging strategies have focused on application-specific flame-retardant requirements by combining MOFs with other N/P-based flame retardants. γ-Propyl-trimethoxysilane (KH550)-functionalized siliceous microencapsulated ammonium polyphosphate (SiAPP-NH_2_) was synthesized via interface modulation and combined with a copper MOF (MOF-Cu) through microencapsulation and electrostatic self-assembly to form SiAPP-NH_2_@MOF-Cu. TPU composites prepared by melt blending with SiAPP-NH_2_@MOF-Cu exhibited excellent flame retardancy and favorable electromagnetic shielding [60]. Figure 5 demonstrates the digital combustion pictures. These results indicate that refined, application-oriented designs that balance flame retardancy with additional functionalities provide a promising route for practical MOF-based flame retardants.

### 3.3. MOFs in Polylactic Acid (PLA) for Fire Retardancy

PLA is a renewable and biodegradable thermoplastic polyester, which plays an important role and holds research value in the field of green materials under the current “dual carbon” goals. However, the flammability, high combustion rate, and low thermal stability of PLA limit its application in practical engineering fields [66,67,68]. Recent research subjects and their corresponding findings are presented in Table 3.

Composites based on ZIFs have been extensively studied for flame retardancy in PLA. A novel degradable flame-retardant system (NanoZIF-8@GO&RDP) was prepared using nano-ZIF-8, GO, and RDP. The material was uniformly dispersed in the PLA matrix, significantly enhancing flame retardancy, with the LOI exceeding 27.0% and achieving a UL-94 VTM-0 rating [69]. A multilayer core–shell flame-retardant system (ZIF-8@CH@APP) was constructed via organic–inorganic hybridization. Bio-based chitosan (CH) was used to functionalize the APP interface, while ZIF-8 was grown in situ to form ZIF-8@CH@APP. This system exhibited excellent flame-retardant performance and good degradability [70].

Zr-based MOFs, with abundant cavities and porosity, can be combined with other materials to form flame-retardant systems. Triethyl phosphate (TEP) was in situ encapsulated in UiO-66-NH_2_ cavities to prepare the composite flame-retardant UiO@TEPx. This system exhibited excellent flame-retardant performance, and its loose, porous structure provided effective smoke suppression [71]. Transition metal-based MOFs (e.g., Co-MOFs, Zn-MOFs, Ce-MOFs) have also been widely applied. Ce-MOFs were synthesized via coordination of cerium oxide with dicarboxylic acids and amino compounds. Multiple hydrogen-bonding interactions between Ce-MOFs and PLA molecules generated a nano-confinement effect, enhancing the flame retardancy of PLA [72]. This study explored the design of MOFs at a microscopic scale, providing a new direction for their application in flame retardancy.

**Figure 6:**
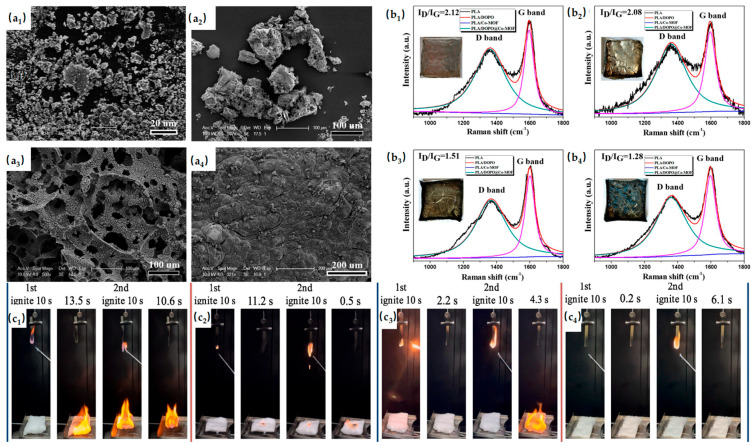
(**a_1_–a_4_**) SEM images of the char residues’ neat PLA (**a_1_**), PLA/DOPO (**a_2_**), PLA/Co-MOF (**a_3_**), and PLA/DOPO@Co-MOF (**a_4_**). (**b_1_**–**b_4_**) The residues’ Raman spectra and digital images of PLA (**b_1_**), PLA/DOPO (**b_2_**), PLA/Co-MOF (**b_3_**), and PLA/DOPO@Co-MOF (**b_4_**). Reproduced with permission from ref. [75]. (**c_1_**–**c_4_**) Photographs documented from UL-94 tests of (**c_1_**) PLA, (**c_2_**) PLA/4M/0A/10 T, (**c_3_**) PLA/0M/4A/10 T and (**c_4_**) PLA/4M/4A/10 T. Reproduced with permission from ref. [73].

Co-based MOFs have been extensively studied. A P&N-rich organic ligand was used to synthesize a Co-MOF with a multilayered mesoporous structure. This Co-MOF was compounded with eTA and APP to form a synergistic flame-retardant system for PLA. Combustion tests demonstrated effective flame retardancy and suppression of melt dripping, while maintaining mechanical properties [73]. Figure 6 demonstrates the photographs. PAPP was combined with ZIF-67 to form a synergistic flame-retardant system. This combination promoted the formation of a high-quality, crack-free intumescent carbon layer, significantly enhancing the protective barrier effect during combustion [74]. Co-MOF was modified with DOPO to form a synergistic flame-retardant system for PLA. The combination enhanced fire safety and also positively influenced the mechanical properties of the composite [75]. Figure 6 demonstrates the SEM images of the char residues. 

The polar functional groups in MOF organic ligands can lead to poor dispersibility in polymer matrices. Functionalization of MOFs with alkali-etched silicon carbide nanoparticles (SiCe) improved their dispersibility in PLA. The resulting PLA composites exhibited excellent mechanical properties and enhanced flame-retardant performance [76]. Improving MOF dispersion in polymer matrices is an effective approach to enhance flame-retardant performance, expanding design strategies for flame-retardant MOFs. Cyclodextrin-based molecules have also been applied in PLA matrices. β-Cyclodextrin (β-CD), with its supramolecular cage-like structure, hydrophobic inner cavity, and external hydroxyl groups, facilitates interactions between guest and host molecules. A tri-functional host–guest flame retardant (β-CD-MOF@Schiff base) demonstrated strong flame-retardant performance at low loading (5 wt%), particularly reducing CO emissions [77]. Research on flame-retardant MOFs in PLA matrices is extensive, with most efforts focused on exploiting MOFs’ compatibility to construct synergistic systems with other flame-retardant materials.

### 3.4. MOFs in Polycarbonate (PC) for Fire Retardancy

PC is a transparent engineering plastic with excellent mechanical strength and impact resistance, and it has numerous applications in engineering fields such as 3D printing, microchips, and batteries. Although PC possesses a certain degree of flame retardancy (with a LOI of 25% and a UL-94 rating of V-2), it melts, drips, or even splatters during combustion and releases toxic gases. Therefore, there is still an inevitable work to ameliorate its flame retardancy in many application scenarios [78,79,80,81,82]. The introduction of MOFs provides a low-smoke and high-efficiency flame-retardant solution for PC. Recent research subjects and their corresponding findings are presented in Table 4.

**Figure 7:**
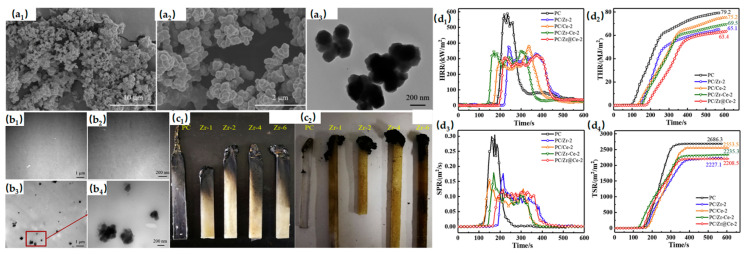
(**a_1_**–**a_3_**) SEM (**a_1_**,**a_2_**) and TEM (**a_3_**) images of Zr-BDC. (**b_1_**–**b_4_**) TEM images of PC (**b_1_**,**b_2_**) and PC/Zr^−4^ (**b_3_**,**b_4_**) at different magnifications. (**c_1_,c_2_**) Photos of samples after UL-94 vertical burning test (**c_1_**) and LOI test (**c_2_**). Reproduced with permission from ref. [83]. (**d_1_**–**d_4_**) HRR (**d_1_**), THR (**d_2_**), SPR (**d_3_**) and TSR (**d_4_**) verse time plots of PC and its composites. Reproduced with permission from ref. [84].

Among MOFs applied as flame retardants in PC, Zr-based MOFs are the most widely used. Zr-BDC with high porosity was synthesized via a solvothermal method and compounded with PC to enhance thermal stability. The formation of a dense, highly graphitized char layer plays a key role in improving flame-retardant performance [83]. Figure 7 demonstrates the SEM and TEM images. Their work provides a new direction for MOF-based synergistic flame-retardant systems. In addition, CeHPP was used as a nanoparticle template to combine the advantages of Zr-BDC and CeHPP. A hybrid composite (Zr-BDC@CeHPP) was prepared and blended with PC, enhancing its thermal stability and fire safety [84]. Figure 7 demonstrates the main flame retardant parameters verse time plots of PC. Their work provides a new direction for MOF-based synergistic flame-retardant systems. In PC/ABS blends, Zr-MOF (UiO-66) was synthesized and applied as a flame-retardant synergist. UiO-66, with its outstanding thermal stability, significantly enhanced the performance of intumescent flame retardants, demonstrating the potential synergistic effect of MOFs in PC/ABS/HPCTP systems and supporting further development of MOFs in PC flame retardancy [85]. Furthermore, compounding MOFs with phosphorus-containing flame retardants has been widely applied in PC. MIL-53(Al) was used to functionalize BP (BP@MIL-53), achieving excellent flame-retardant performance at a low loading of 1.0 wt% [86]. Meanwhile, modifying black phosphorus with MOFs expands the range of MOF-based synergistic flame-retardant materials and provides novel and scalable ideas for the design of flame-retardant MOFs.

### 3.5. MOFs in Polyethylene Terephthalate (PET) for Fire Retardancy

PET is an unsaturated polyester material composed of elements such as carbon, hydrogen, and oxygen. As a thermoplastic polyester with excellent mechanical properties, it is resistant to oils, fats, dilute acids, and dilute alkalis. Additionally, it can be processed into polyester fibers (terylene). Therefore, PET has extensive applications in fields such as electronics and electrical appliances, plastic containers, and films [87,88,89,90]. Recent research subjects and their corresponding findings are presented in Table 5.

**Figure 8:**
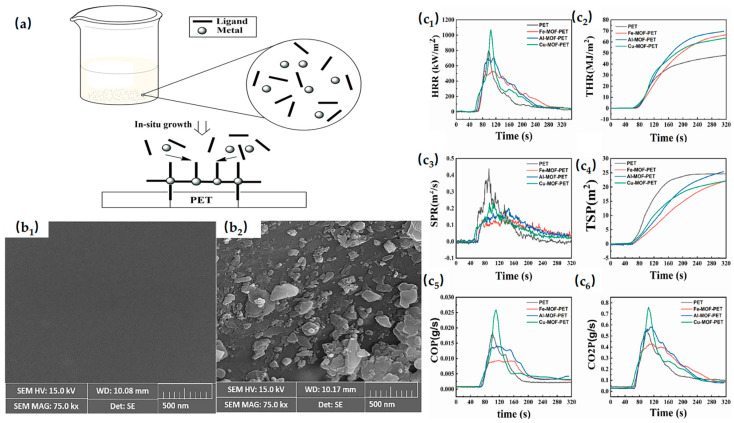
(**a**) Schematic Diagram of MOF Growth on PET Substrate. (**b_1_,b_2_**) SEM image of untreated samples (**b_1_**) and ZnO based MOF treated samples (**b_2_**). Reproduced with permission from ref. [93] (**c_1_**–**c_6_**) P CONE test results of PET and MOF-PET: HRR curves (**c_1_**); THR curves (**c_2_**); SPR curves (**c_3_**); TSP curves (**c_4_**); CO production curves (**c_5_**); and CO_2_ production curves (**c_6_**). Reproduced with permission from ref. [91].

There have been numerous systematic research studies and experiments on transition metal-based MOFs in PET. MOFs were synthesized using Fe^3+^, Al^3+^, and Cu^2+^ with terephthalic acid ligands and incorporated into PET to prepare Fe-MOF/PET, Al-MOF/PET, and Cu-MOF/PET composites. Combustion tests showed that the flame-retardant performance followed the order: Fe > Al > Cu [91]. Figure 8 demonstrates the P CONE test results of PET and MOF-PET. This study provides a foundation for understanding the flame retardancy of MOFs with typical metal cations and guides the design of MOF-based flame retardants. The iron-based MOF MIL-88B(Fe) was introduced into PET, and combustion tests showed significantly improved flame retardancy, primarily due to the formation of highly graphitized, rigid, non-flammable char residues catalyzed by MIL-88B(Fe) [92].

However, during combustion, PET generates benzoic acid and its precursors, which pose a risk of heavy smoke emission. MOFs were synthesized in situ on textile products to impart multifunctional properties. Polyester fabrics were activated via alkaline hydrolysis, followed by growth of ZnO-based MOFs on the surfaces. Combustion tests showed significant flame-retardant effects, while the MOF-treated fabrics also exhibited increased tensile strength, thickness, and stiffness [93]. Figure 8 demonstrates the SEM image. Research on flame-retardant MOFs in PET matrices remains limited, largely due to PET’s high crystallinity and rigid chain segments, which promote MOF agglomeration. Strategies such as in situ MOF growth have been employed to improve dispersion. Despite these advances, the application of MOFs in PET still faces significant challenges and considerable potential for further research.

### 3.6. Flame-Retardant Application of MOFs in Polystyrene (PS)

PS is a colorless and transparent thermoplastic polymer synthesized from styrene monomers. It exhibits excellent optical transparency, electrical insulation, and processability, and is therefore widely used in disposable tableware, food packaging, foam insulation materials, and household appliance casings. However, the application of PS in high-temperature environments is severely limited by its relatively low thermal resistance. The glass transition temperature of PS is typically around 95–105 °C, beyond which the material undergoes significant softening and dimensional deformation. Moreover, thermal degradation of PS generally occurs at temperatures above 300 °C, accompanied by the release of volatile and potentially harmful decomposition products. These thermal limitations significantly restrict the use of PS in high-temperature and engineering applications [94,95,96,97,98]. Recent research subjects and their corresponding findings are presented in Table 6.

Polystyrene (PS) is characterized by high heat release rates, low thermal stability, poor char formation, and pronounced molten dripping during combustion. These features make it highly flammable and difficult to self-protect [101,102]. During the pyrolysis of the PS matrix, gaseous products released from the MOFs reduce the concentration of degradation products and reactive oxygen species, while the resulting porous metal oxides act as effective barriers to heat and fuel transfer. The synergistic action of these mechanisms contributes to the superior thermal stability and flame retardancy of the PS/MOFs composites.

**Figure 9:**
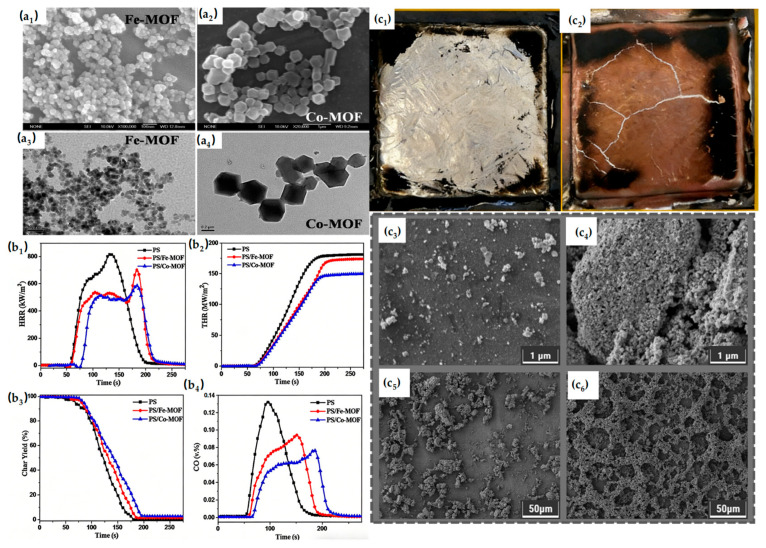
(**a_1_**–**a_4_**) SEM (**a_1_**,**a_2_**) and TEM (**a_3_**,**a_4_**) images of MOFs. (**b_1_**–**b_4_**) HRR (**b_1_**), THR (**b_2_**), weight loss (**b_3_**), and CO (**b_4_**) curves of PS and its composites obtained by cone calorimetry. Reproduced with permission from ref. [94] (**c_1_**–**c_6_**) Digital pictures of char residues of (**c_1_**) PS; (**c_2_**) PS/T-Fe-3.0. SEM images of char residues of (**c_3_**,**c_4_**) PS and (**c_5_**,**c_6_**) PS/T-Fe-3.0. Reproduced with permission from ref. [100].

Transition metal-based MOFs are widely studied as flame retardants for PS. Cobalt-based MOFs synthesized via a solvothermal approach effectively enhanced the thermal stability of PS composites. Analysis of TGA-FTIR data and combustion residues suggests that the synergistic interaction between the MOFs’ thermal barrier effect and catalytic action improves flame retardancy. Incorporation of MOFs enhances fire safety and offers potential for the development of fire-resistant polymer materials [94]. Figure 9 demonstrates the SEM and TEM images of MOFs. To address heavy smoke release during PS combustion, Zr-MOF (UiO-66) was synthesized via a solvothermal method and incorporated into PS. UiO-66 exhibited good dispersibility and compatibility in the PS matrix, enhancing both flame retardancy and smoke suppression. Analysis of residues and pyrolysis products indicated that UiO-66 promotes carbonization and thermal barrier effects, providing guidance for further research on UiO-66 in polymer systems [99]. Zhao et al. considered the influence of MOF pore size on its flame-retardant performance. They attempted to utilize the microporous MIL-53 to develop a macroporous MOF (P-Fe-MOF) and then used its excellent porous structure to adsorb TEP as a flame retardant for PS, thereby reducing its fire hazard. Through XPS analysis, TEP uniformly distributed in P-Fe-MOF was confirmed. Subsequent analysis of the combustion performance of the PS composites showed that the prepared PS composites had better flame retardancy than pure PS [100]. Figure 9 demonstrates the digital pictures and SEM images of char residues. This work provides strategies for adjusting MOF pore size and preparing novel macroporous MOFs, offering effective approaches for MOF-based flame-retardant research. Co-based MOF-71-NH_2_ was synthesized via post-synthetic modification (PSM) and further modified with PCT to form a synergistic flame-retardant system (PCT@MOF-NH_2_). Combustion tests demonstrated significantly enhanced fire safety in PS, and analysis of gaseous and condensed products indicated that the flame-retardant mechanism is primarily attributable to the barrier effect of PCT@MOF-NH_2_, expanding the application prospects of MOFs in polymer flame retardancy [38].

## 4. Summary and Outlook

MOFs are driving a transformative impact in materials science due to their unique structural characteristics. The 2025 Nobel Prize in Chemistry recognized key contributors in the MOF field, highlighting the transition of MOFs from fundamental research to industrial applications, including flame retardancy. This review summarizes the progress of MOFs as flame-retardant additives in thermoplastic polymer composites, systematically covering their application in six widely used polymers: PP, TPU, PLA, PC, PET, and PS. MOFs and their derivatives have only recently been explored as novel flame retardants, particularly in thermoplastic matrices, indicating substantial opportunities for further research and development.

The flame retardancy of MOFs is basically inseparable from their loose and porous structure. On the one hand, this structure can absorb and catalyze the degradation products generated during polymer combustion, thereby densifying the formed char layer, enhancing its barrier effect on flame spread and the transfer of pyrolysis products, and reducing the emission of toxic gases and smoke. On the other hand, the porous structure of MOFs also endows them with excellent processability. MOFs can exhibit a synergistic effect with traditional inorganic flame retardants, organic flame retardants, and reactive flame retardants to enhance their flame-retardant performance and often can also impart additional properties to the system. MOF products always have an elaborate internal structure, which contributes to their powerful and diverse properties. It can even be said that MOFs will never become outdated in future research: they can be compounded with other materials to achieve synergy; their inorganic metal centers and organic ligands possess extremely rich variability; and there is still considerable research value and application potential to be explored.

Nevertheless, research on flame-retardant metal–organic frameworks (MOFs) remains in its early stages. In thermoplastic matrices, challenges such as poor MOF dispersion, limited interfacial compatibility, and high production costs persist. Experimental studies indicate that MOFs alone rarely achieve outstanding flame-retardant performance. Most current approaches focus on constructing synergistic systems, including functionalizing MOFs with other materials, combining MOFs with conventional flame retardants, or utilizing MOFs’ large pore volumes to load additional agents. Future research directions include improving interfacial bonding and dispersion through surface modification or functional ligand design; developing diverse MOF-derived flame retardants, such as carbonized MOFs or metal oxide@MOF hybrids; and exploring novel synthesis or incorporation strategies, including in situ growth or sol–gel methods, to achieve controlled morphologies and dispersion states in polymers. Advances in macromolecular synthesis and composite technologies are expected to further enhance flame retardancy in thermoplastic polymers, supporting the transition from laboratory research to industrial applications.

With the 2025 Nobel Prize in Chemistry awarded to outstanding scientists in MOF-related fields, MOFs will undoubtedly attract broader attention and spark a surge in research. It can be anticipated that in the future, more novel functional ligands will be designed and synthesized, while an increasing number of metal centers will be explored for constructing diverse MOF structures. This will not only greatly enrich the structural types and functional diversity of MOFs but also lay a solid foundation for their applications in multiple cutting-edge fields such as gas storage, catalysis, drug delivery, separation, and flame retardancy. In the field of flame retardancy, although the development of high-efficiency and environmentally friendly MOF-based flame retardants still faces numerous challenges, each systematic structural design, performance optimization, and mechanism exploration will bring valuable experience and innovative ideas to materials science and chemical engineering. It is foreseeable that the continuous in-depth research in this field will not only promote the development of new flame-retardant materials but also provide important support for achieving sustainable development goals. Therefore, the research on MOFs has broad prospects, and its inherent scientific value and application potential will undoubtedly inspire more researchers to continuously explore and scale new heights in this field.

## Figures and Tables

**Table 2:** Test data of flame-retardant performance of thermoplastic polyurethane (TPU) with different MOFs.

MOF Flame-Retardant System	Flame-Retardant Results	References
UIO-66 (Zr)	2% usage, 26% of LOI; UL-94 V-1	[54]
NH_2_-UIO-66 (Zr)	2% usage, 26% of LOI; UL94 V-1	[54]
SiO_2_ functionalized NH_2_-UIO-66 (Zr)	LOI 26% and UL94 V-1	[54]
APP@NH_2_-UIO-66 (Zr)	75.76%, 86.19%, 69.74% and 86.34% reduction in pHRR, THR, SPR and TSP	[55]
ZIF-67 H	2 wt% addition, 45.4%, 50.2%, and 72.9% reduction in pHRR, TSP, and SF	[56]
ZIF-67H@PBA	0.5 wt% addition, 33.6%, 47%, and 61% reduction in pHRR, TSP, and SF	[56]
Ni-MOF@TiC_2_T_x_	1 wt% addition, 15%, 44%, 26%, 18%, and 8% reduction in HRR, SPR, TSR, CO production, and CO_2_ production	[57]
La-MOF@MXene	3 wt% addition, 49.4% reduction in pHRR, while TSP, CO production and CO_2_ production were also reduced.	[58]
MXene@SiC/ZIF67@PANI	71.4% and 34.6% reduction in pHRR and THR; 26.2% of LOI	[59]
SiAPP-NH2@MOF-Cu	78.7%, 51.3%, 59.3%, and 58.7% reduction in pHRR, THR, TSP, and CO_2_ production	[60]

**Table 3:** Test data of flame-retardant performance of polylactic acid (PLA) with different MOFs.

MOF Flame-Retardant System	Flame-Retardant Results	References
NanoZIF-8@GO&RDP	27.0% of LOI; UL94 VTM-0	[69]
ZIF-8@CH@APP	7 wt% addition, 0.2 m^2^ reduction in TSP; UL-94 V-0	[70]
UiO@TEP_x_	2% content, UL-94 V-0, 24.2% of LOI, 29.5% and 14.2% reduction in TSP and PHRR	[71]
CeC_x_O_y_-P-3	27.8% and 80% reduction in PHRR and the peak CO release	[72]
Co-MOFs@APP&eTA	27.0% of LOI; UL-94 V-0	[73]
ZIF-67@PAPP	0.06 wt% ZIF-67 and 4.96 wt% PAPP, UL-94 V-0	[74]
DOPO@Co-MOF	27%, 56%, and 20% reduction in PHRR, PSPR, and total CO yields	[75]
SICe@MOFs	33.9%, 43.8%, 35.7%, and 24.1% reduction in pHRR, THR, CO production, and CO_2_ production	[76]
β-CD-MOF@Schiff base	15%, 17%, and 62% reduction in pHRR, THR and total CO yields; 29% of LOI	[77]

**Table 4:** Test data of flame-retardant performance of polycarbonate (PC) with different MOFs.

MOF Flame-Retardant System	Flame-Retardant Results	References
Zr-BDC	4 wt% addition, 48% and 34% reduction in PHRR and TSR; UL-94 V-0	[83]
Zr-BDC@CeHPP	2 wt% addition, 45%, 20%, 74%, and 18% reduction in PHRR, THR, PSEA, and TSR; UL-94 V-0	[84]
Uio-66&HPCTP	7.0 wt% HPCTP and 3.0 wt% UiO-66, 27.0% of LOI; UL-94 V-0	[85]
BP@MIL-53	1.0 wt% addition, 49.4% and 19.3% reduction in pHRR and THR; 30.5% of LOI; UL-94 V-0	[86]

**Table 5:** Test data of flame-retardant performance of polyethylene terephthalate (PET) with different MOFs.

MOF Flame-Retardant System	Flame-Retardant Results	References
Fe-MOF	28% of LOI; UL94 V-2	[91]
Al-MOF	25% of LOI; UL94 V-2	[91]
Cu-MOF	24% of LOI; UL94 V-2	[91]
MIL-88B (Fe)	27% of LOI; UL94 V-0	[92]
ZnO-MOFs	Compared to raw sample, ash from 1% to 9%, residue at 600 °C from 0.66% to 1.16%.	[93]

**Table 6:** Test data of flame-retardant performance of polystyrene (PS) with different MOFs.

MOF Flame-Retardant System	Flame-Retardant Results	References
Fe-MOF	14.8% and 4.0% reduction in pHRR and THR	[94]
Co-MOF	28.0% and 17.6% reduction in pHRR and THR	[94]
UiO-66	5 wt% addition, 26.8% and 14.7% reduction in pHRR and THR	[99]
P-Fe-MOF&TEP	24.7% and 12.7% reduction in pHRR and THR	[100]
PCT@MOF-NH2	40% and 31% reduction in pHRR and THR	[38]

## Data Availability

No new data were created or analyzed in this study. Data sharing is not applicable to this article.

## Abbreviations

The following abbreviations are used in this manuscript:

APP	Ammonium Polyphosphate
BP	Black Phosphorus
CeHPP	Cerium Phenylphosphonate
CTAB	Cetyltrimethylammonium Bromide
DOPO	9,10-Dihydro-9-oxa-10-phosphaphenanthrene-10-oxide
EP	Epoxy
FRs	Flame Retardants
HCCP	Hexachlorocyclopentadiene
HPCTP	Hexaphenoxycyclotriphosphazene
IFR	Intumescent Flame Retardants
KH550	γ-Aminopropyltriethoxysilane
LOI	Limited Oxygen Index
MMT	Montmorillonite
MOF	Metal–Organic Framework
PANI	Polyaniline
PAPP	Piperazine Pyrophosphate
PBA	Polybutylene Adipate
PC	Polycarbonate
PET	Polyethylene Terephthalate
PLA	Polylactic Acid
PP	Polypropylene
PCT	Phosphonitrile Chloride Trimer
pHRR	Peak Heat Release Rate
PS	Polystyrene
PSM	Post-Synthetic Modification
pSPR	Linear Dichroism
PUA	Three Letter Acronym
RDP	Resorcinol Bisdiphenyl Phosphate
THR	Total Heat Rejection
TPU	Thermoplastic Polyurethane
TSR	Linear Dichroism
UPR	Unsaturated Polyester Resin
XPS	X-ray Photoelectron Spectroscopy
β-CD	β-Cyclodextrin
